# Rectal Stimulation in Premature and Full-Term Newborns: A Narrative Review

**DOI:** 10.3390/children12121656

**Published:** 2025-12-06

**Authors:** Silvia Rossi, Simona Calza, Chiara De Rosa, Giulia Ottonello, Nicoletta Dasso, Roberta Da Rin Della Mora, Ilaria Artuso, Giuseppe Minniti Caracciolo, Simona Serveli, Silvia Scelsi

**Affiliations:** Health Professional Direction, IRCCS Istituto Giannina Gaslini, Largo G. Gaslini 5, 16100 Genoa, Italy; silviarossi@gaslini.org (S.R.); chiaraderosa@gaslini.org (C.D.R.); giuliaottonello@gaslini.org (G.O.); nicolettadasso@gaslini.org (N.D.); robertadarindellamora@gaslini.org (R.D.R.D.M.); ilariaartuso@gaslini.org (I.A.); giuseppeminniti@gaslini.org (G.M.C.); simonaserveli@gaslini.org (S.S.); silviascelsi@gaslini.org (S.S.)

**Keywords:** rectal stimulation, review literature, premature, preterm, newborn, nursing care

## Abstract

**Highlights:**

**What Is Already Known**

- We currently know that the nursing care practice of rectal stimulation could lead to adverse outcomes in the newborn population.
- Currently, there are no guidelines available for performing this practice safely.

**What This Paper Adds**

- This review highlights the urgent need for studies to inform national and interna-tional guidelines and procedures to ensure the safety and quality of rectal stimulation nursing procedures for preterm and full-term newborns.
- This review could directly impact the practice of rectal stimulation because it is cur-rently performed and taught to newborn caregivers by healthcare professionals.

**Abstract:**

Purpose: Nurse professionals often practice rectal stimulation in a hospital setting to help premature or full-term babies evacuate or to avoid abdominal pain caused by gas colic. Paediatricians also recommend this technique to newborn caregivers, who can carry it out independently at home. To the best of our knowledge, there are no established national guidelines on how to implement this technique safely or what the clinical/care indications are for its use. Our purpose is to synthesise the evidence on the nursing practice of rectal stimulation in preterm and full-term newborns. Methods: A narrative literature review was conducted. A search was conducted across six databases in February, followed by a manual review of the included studies’ bibliographies, and another search in October 2023. The search strategy identified studies published without date limits. The articles were screened according to the inclusion criteria. Results: Sixty-two articles were retrieved. Following the screening process, only two articles were selected for inclusion in the final synthesis. Both studies evaluated the effects of enemas and/or rectal stimulation on feeding tolerance and bowel habits in preterm newborns. Even if both studies included information on when rectal stimulation in preterm newborns is indicated and how it is conducted, they are insufficient to provide a clear statement for nursing practice. Conclusions: Nowadays, nurses perform rectal stimulation and teach it to caregivers of newborns without shared international or national guidelines or procedures focused on patient safety. It is desirable to conduct scientific studies to inform nursing practice and enhance the quality of nursing care delivered. This review also highlights a critical gap in evidence regarding the use of rectal stimulation in full-term newborns and in community settings.

## 1. Background

In high-income countries, the survival of preterm newborns is constantly increasing, with near-universal survival for those born after 28 weeks of gestation, thanks to advancements in medical, nursing, and technological sciences in this field [1]. However, significant inequalities in care persist both between and within countries, contributing to unacceptably significant survival gaps for preterm infants. Globally, while over 13 million preterm newborns survive each year, many still require developmentally supportive care to ensure healthy growth and well-being, with up to 2.7% experiencing moderate to severe impairments and 4.4% experiencing mild neurodevelopmental issues. This progress emphasises the need for neonatal nurses to be highly specialised in delivering safe, evidence-based care [2,3]. One of the first needs that arises is the delivery of evidence-based nursing care, which involves providing nursing care that focuses on patient safety and quality of care [3,4]. The quality of nursing care can be enhanced by establishing structured guidelines and procedures that improve nursing care outcomes [5].

Within the complex care needs of preterm newborns, gastrointestinal immaturity often results in delayed meconium passage, reduced bowel motility, and feeding intolerance. These challenges necessitate targeted bowel management strategies to facilitate adequate evacuation and minimise discomfort. Among the various empirical practices used daily in neonatal care units, one such intervention is rectal stimulation via a catheter [6]. This practice is also commonly performed on full-term newborns and taught to caregivers, particularly in Italian neonatal settings, as a strategy to relieve presumed abdominal discomfort at home. In the first hours or days of life, rectal stimulation is sometimes used to promote meconium passage in both preterm and full-term infants—an aspect that is only partially addressed in the literature [7]. However, its use in otherwise healthy infants at home is empirical mainly and not supported by robust scientific evidence. Despite its widespread use, no clear guidelines exist to support this practice in the home setting.

Preterm newborns are often affected by a reduction in bowel motility, caused by the immaturity of their gastrointestinal system, and the resultant faeces or air stasis in the abdomen, associated with difficulties in evacuation, can cause discomfort, pain, inconsolable crying, a distended abdomen, and feeding intolerance. In addition, the prolonged presence of faeces in the abdomen exposes this population to an increasing risk of feeding intolerance [8], and gastrointestinal dysmotility exposes them to a rising risk of necrotising enterocolitis [9].

Although performed empirically, the effectiveness and safety of rectal stimulation remain insufficiently supported by scientific evidence. Additionally, during the newborn’s hospital stay, this practice is taught to caregivers, who then frequently apply it at home to reduce pain associated with intestinal colic. Other empirical evidence suggests that this practice helps maintain an adequate sleep–wake rhythm, as reduced pain symptoms would facilitate the newborn’s sleep. However, such procedures, such as enema administration, if not performed with specific attention and expertise, have been associated with substantial adverse outcomes, including bowel perforation [10,11].

Given the frequent use of this practice in NICUs and at home, and the absence of standardised guidelines ensuring safety, we have decided to conduct a narrative literature review that can answer our research question: what are the clinical and care indications for the performance of rectal stimulation via a single-use catheter in a preterm or full-term newborn? Moreover, what are the correct methods and execution timing?

## 2. Aim

This narrative review aims to provide an overview and synthesis of the current evidence on rectal stimulation in preterm and full-term newborns, identify the clinical and care indications for performing this practice, and outline appropriate methods in terms of efficacy and safety.

To our knowledge, this is the first review that addresses this topic.

## 3. Methods

### 3.1. Design

The present study is a narrative review of published research evidence; therefore, ethical approval was not required, and the protocol was not registered.

We decided to conduct a narrative review because it allows us to evaluate the current state of published research on a specific topic. It differs from systematic reviews because narrative reviews employ a different methodology, focusing on finding synthesis and aiming to answer broader, general questions. Additionally, the narrative review identifies new areas of study that have yet to be addressed [12].

No acknowledged guidelines are available for writing narrative reviews; therefore, we applied the SANRA scale (Scale for the Assessment of Narrative Review Articles) to ensure quality and transparency in reporting [13] (see Supplementary File S1).

### 3.2. Search Methods

The search strategy was developed in consultation with a health sciences librarian. Initially, we tested multiple combinations of keywords and subject headings—including synonyms and related terms such as “anal stimulation”, “digital rectal stimulation”, “rectal tube”, “rectal probe”, “enema”, “bowel management”, and “meconium passage”—but these attempts yielded no relevant results. Therefore, we refined and simplified the search strategy to “rectal stimulation” AND (“newborn” OR “premature” OR “preterm”). The final search strings for each database are reported in Supplementary File S2.

The search strategy was applied to PubMed, CINAHL, Scopus, Cochrane, Embase, and Web of Science databases. The initial searches covered the period from database inception to February 2023 and were launched in February 2023. Subsequently, due to the paucity of studies retrieved, an updated search was launched in October 2023 to identify new studies of interest published between March 2023 and October 2023.

Additionally, we conducted an exploratory search of grey literature sources (e.g., Google Scholar, national/institutional guidelines, professional nursing and neonatology associations), which confirmed the absence of published protocols or recommendations on the practice of rectal stimulation in preterm and newborn infants.

After duplicates were removed, two research team members screened study titles, abstracts, and full-text articles. They then hand-searched the reference lists of included studies to identify any additional eligible studies.

Any inconsistencies regarding article relevance for inclusion at the full-text screening stage were solved through discussions between the reviewers.

To better assess the methodological rigour of the included studies, we conducted a structured critical appraisal using the Joanna Briggs Institute (JBI) checklists, one for randomised controlled trials and another for analytical cross-sectional studies [14,15].

### 3.3. Inclusion and Exclusion Criteria

Studies were included if they met the following inclusion criteria: the sample consisted of preterm or full-term newborns, the studies were published in English or Italian, and they were primary studies (including case reports and conference abstracts) that focused on rectal stimulation.

Exclusion criteria included studies on surgical procedures or diagnostic techniques, as well as studies involving patients with gastrointestinal abnormalities.

No publication date limits were applied.

### 3.4. Data Abstraction and Synthesis

Three authors developed a data extraction form with the following fields: author, year and country, aim, design and sampling, intervention, results, a clinical indication for rectal stimulation, and details of rectal stimulation (e.g., probe diameters, guidelines followed, etc.).

Two authors independently extracted the data and completed the form using information retrieved from the included studies.

The characteristics of all studies were summarised to provide a narrative synthesis that addresses the review’s aim.

## 4. Results

The final search (launched in October 2023) resulted in 62 articles through database searching. After 39 duplicates were removed, twenty-three articles remained. These were assessed for eligibility through abstract screening. At the end of the process, 19 articles were removed, and 4 underwent full-text screening. Two articles were removed at the end of full-text screening because they were unrelated to rectal stimulation.

No studies that met our criteria were identified through hand-searching reference lists.

At the end of the process, only two articles met the inclusion criteria and were included in the narrative synthesis (see Supplementary File S3, Prisma Flow Diagram).

### 4.1. Characteristics of Included Studies

The two studies included were published in 2012 and 2013 and are from the same research group. The first is an observational study, and the second is a randomised controlled trial. Both were conducted in Spain and enrolled preterm newborns under 28 weeks of gestational age. Both studies evaluated the effect of enemas and/or rectal stimulation on feeding tolerance and bowel habits in different ways (see Table 1 for more details). The observational study [16] aimed to determine whether the acquisition of normal bowel habits (facilitated by rectal stimulation) is associated with better growth and earlier attainment of full enteral feeding. The RCT study [17] focuses on the association between rectal stimulation and enema use and a normal stooling pattern.

### 4.2. Quality Appraisal of Included Studies

Overall, both studies were classified as of moderate methodological quality: the randomised controlled trial met 8 out of 13 criteria. Three criteria were not met: participant blinding was not feasible due to the nature of the intervention, and the analysis did not report whether outcomes differed between those who completed follow-up and those who did not. Two items were unclear, particularly regarding allocation concealment and blinding of outcome assessors.

The observational study met six out of eight criteria. It clearly defined the study population and outcome measures and used appropriate statistical analysis. However, it was unclear how potential confounding factors were identified and managed.

### 4.3. Synthesis of the Studies

Both studies investigated the use of rectal stimulation in extremely preterm newborns and provided procedural details and clinical indications for its use.

In the observational study [16], rectal stimulation was performed using a lubricated CH8 bladder catheter inserted approximately 1.5–2 cm into the rectal ampulla, with slow circular movements maintained for 15–30 s.

In the RCT study [17], a single-use catheter coated with vaseline was used, inserted to a similar depth; however, no details were provided regarding its size, intended use, or duration of application.

Both studies applied the same clinical indications: rectal stimulation was performed in cases of abdominal distension or the absence of defecation in the previous 24 h. However, neither study defined how abdominal distension was evaluated, and key outcomes, such as feeding tolerance or stool passage, were not operationalised or measured using standardised criteria.

Moreover, no quantitative effect estimates were reported in either study, making it difficult to assess the clinical relevance of the observed effects.

## 5. Discussion

This narrative literature review aimed to explore the clinical and care indications for rectal stimulation via catheter in preterm and full-term newborns and to assess how this procedure is currently performed, including its safety and efficacy. Despite being a common nursing practice in many Italian NICUs—and occasionally taught to caregivers for home use—there is a striking lack of scientific evidence to guide its application.

The results of this review have revealed a substantial lack of published scientific evidence, because only two small studies met the inclusion criteria, highlighting the limited evidence base.

The two studies included in this review, both conducted by the same research group in Spain and limited to extremely preterm infants (<28 weeks GA), do not provide sufficient evidence to support or standardise clinical practice. Notably, both studies lack standardised definitions for key outcomes such as feeding tolerance, meconium passage, and abdominal distension, and no quantitative effect estimates were reported. Moreover, the co-occurrence of rectal stimulation and enemas in both protocols makes it challenging to isolate the effects of rectal stimulation alone.

Preterm newborns display delayed spontaneous meconium evacuation compared to full-term newborns. They require more frequent nutritional support via parenteral or enteral lines. In newborns, the ability to contract and release the anal sphincter to evacuate is not entirely acquired. In addition, preterm newborns often receive non-invasive respiratory support, such as high-flow nasal cannula (HFNC) or nasal continuous positive airway pressure (nCPAP), which can lead to abdominal distension from air swallowing or gas accumulation in the gastrointestinal tract. All of these conditions could lead to abdominal discomfort [18] that is often treated with rectal stimulation via a single-use catheter.

The literature shows that rapid meconium evacuation in preterm newborns is a crucial factor that promotes feeding tolerance, with the amount of feeding tolerated inversely related to the rate of meconium evacuation: the more rapid the meconium evacuation, the more feeding the newborn tolerates. On the other hand, delayed meconium evacuation is associated with reduced tolerance to feeding [19]. The achievement of regular bowel habits is linked to full enteral feeding and reduced time spent on parenteral nutrition support. Parenteral nutrition is administered via a central venous catheter, so the early achievement of full enteral feeding is correlated with a lower risk of hospital infection [20,21]. In addition, full enteral feeding, especially with human milk, reduces the risk of malnutrition and increases the weight gain of preterm newborns [22,23].

All of these factors underscore the importance of evacuation stimulation in both preterm and full-term newborns. While rapid meconium evacuation has been associated with earlier enteral feeding and better growth outcomes in preterm infants, there is no consensus on how best to promote such evacuation. Enemas, although commonly used, have shown limited effectiveness and are associated with potential harms in low-birth-weight infants [24], although some recent studies have shown some clinical benefit related to enemas performed with breast milk [25]. Rectal stimulation via catheter is often performed empirically, based on clinical experience rather than scientific validation.

Despite limited evidence, rectal stimulation remains a common clinical practice in many NICUs. Everyone who has even just passed through an Italian NICU has witnessed this practice, which occurs several times a day.

This observation extends beyond Italy; for instance, a German study found that almost 53% of healthcare personnel (doctors and nurses) have used rectal stimulation via a catheter [26].

Given the absence of robust evidence, it is crucial to frame this intervention with explicit caution, especially when considering its routine use or teaching to caregivers. Rectal stimulation must be clearly differentiated from enemas or rectal tube insertion, which may involve different mechanisms and risks. Plausible risks associated with rectal stimulation include mucosal injury, rectal bleeding, infection, and pain or stress for the newborn. The procedure should be strictly avoided in conditions such as suspected or confirmed necrotising enterocolitis (NEC) [27].

In light of these findings, we strongly recommend that healthcare professionals avoid promoting or performing rectal stimulation in routine practice, especially in the absence of defined clinical indications and shared safety protocols. National and international neonatal care bodies should prioritise developing consensus statements and promoting multicentre studies to determine the safety, efficacy, and best practices of this intervention. Until robust data are available, alternative and non-invasive methods—such as abdominal massage or position changes—should be preferred. Moreover, institutions should revise internal protocols and training content to reflect the current lack of evidence and to prevent the transmission of potentially unsafe empirical practices to caregivers.

Based on our findings, future research should prioritise several critical areas. First, there is a need for randomised controlled trials comparing rectal stimulation with non-invasive alternatives such as abdominal massage in preterm infants. Equally important are observational studies assessing the safety and frequency of this practice in full-term infants, particularly when performed at home by caregivers. Further studies should aim to define standardised clinical indications—such as abdominal distension or delayed meconium passage—and outcome measures, such as feeding tolerance. Safety-focused investigations are also needed to examine potential adverse effects, including mucosal injury, rectal bleeding, and neonatal stress responses. Additionally, research efforts should focus on developing and testing clinical guidelines or protocols to ensure safe implementation in both hospital and home settings. Finally, qualitative research exploring healthcare professionals’ and caregivers’ perspectives may help identify barriers and facilitators to evidence-based practice in this area.

Some activities in the health sciences are often performed on the basis of expertise rather than evidence. This is a widespread reality, even if it is hazardous in terms of quality of care and patient safety. This occurs because many healthcare science practices are accepted based on their logic, value, and efficacy, as demonstrated over the years [28]. These practices are therefore performed without any studies or scientific analysis of their effectiveness. At the core of these practices is always a rational, logical flow of decisions that justifies their performance. This could generate confusion, making it unclear what to do when scientific evaluations are missing.

Well-designed studies, including prospective trials and observational data, are urgently needed to assess the effectiveness and safety of rectal stimulation in different neonatal populations. Comparative studies with other non-invasive techniques, such as abdominal massage, may also help to define best practices. Only then will it be appropriate to consider developing evidence-based protocols that can be implemented safely in both clinical and home settings.

This review highlights a significant gap between common neonatal nursing practices and available scientific evidence. By exposing this inconsistency, the present study contributes to a necessary reflection within the nursing and neonatology communities and lays the groundwork for further research and safer clinical protocols.

### Limitations

Although a structured and systematic search strategy was employed in this review, several limitations remain. First, only two studies met the inclusion criteria, both from the same research group and setting, which limits the generalizability of the findings. Second, the included studies did not report standardised outcome definitions or effect estimates, which further limited their interpretability. Finally, restricting the search to English and Italian may have led to the exclusion of relevant studies published in other languages.

## 6. Conclusions

In conclusion, this review found insufficient evidence to draw firm conclusions on the safety and efficacy of rectal stimulation in neonates.

The studies analysed do not specify standardised or evidence-based methods for performing rectal stimulation, and there is no information on the clinical conditions that justify the use of a rectal catheter for stimulation.

In particular, no evidence is currently available for full-term newborns or for home-based practice, despite their frequent involvement in current clinical routines.

The literature, however, highlights the importance of facilitating rapid meconium evacuation, especially in preterm newborns.

These results suggest the need for further studies on this topic, focusing on the technique’s efficacy and on how to perform it safely. This will enable the development of protocols, procedures, or guidelines that are suitable and usable in both hospital settings and at home by caregivers.

Until such evidence becomes available, the routine use of rectal stimulation—particularly in full-term infants or in the home setting—should be approached with extreme caution. We recommend that healthcare professionals refrain from teaching or encouraging this practice without clear clinical indications and safety parameters. National and international neonatal care organisations should promote the development of guidelines and encourage multicentre research initiatives to address this significant gap.

The topic investigated represents a grey area in nursing research, for which further studies are needed. Addressing this knowledge gap is not only a scientific necessity but also an ethical imperative to ensure safe, high-quality, and evidence-informed care for one of the most vulnerable patient populations.

## Figures and Tables

**Table 1:** Data extraction of included studies.

de Pipaón Marcos et al. [16]	Prospective observational study; 121 preterm newborns (GA 23–27 + 6 weeks).	Standardised protocol: rectal stimulation if no defecation in previous 24 h or abdominal distension. If ineffective, enema after 8 h.	Earlier establishment of a normal stooling pattern was associated with earlier full enteral feeding and reduced postnatal undergrowth.	- Abdominal distension (no defined) - No defecation in previous 24 h.	Lubricatedbladder catheter of CH. 8; inserted 1.5–2 cm; circular slow movement for 15–30 s.	Outcomes not clearly defined; “abdominal distension” not operationalized; no effect estimates reported
de Pipaón Marcos et al. [17]	Open RCT; 49 preterm newborn (GA ≤ 28 weeks)	Rectal stimulation every 12 h + 2 enemas/day.	No effect of prophylactic rectal stimulation + enemas on bowel habit normalisation.	Same as above	Single use Vaseline-coated catheter; inserted 1.5–2 cm; catheter type and duration not specified	Outcomes not clearly defined; no standardised criteria; no effect estimates reported.

## Data Availability

Data sharing is not applicable.

## Supplementary Materials

The following supporting information can be downloaded at: https://www.mdpi.com/article/10.3390/children12121656/s1, Supplementary File S1: Scale for the Assessment of Narrative Review Articles—SANRA; Supplementary File S2: Search Strategy; Supplementary File S3: PRISMA 2020 flow diagram for new systematic reviews which included searches of databases and registers only [29].
