# Supplementary material for: Rectal Stimulation in Premature and Full-Term Newborns: A Narrative Review
Source: Children (Basel). 2025 Dec 6;12(12):1656. doi: 10.3390/children12121656 (PMC12731925; doi:10.3390/children12121656)
Supplement: Supplementary file 1 [file children-12-01656-s001.zip › Supplementary File S2.pdf]

Supplementary File S2. ***Search Strategy***

DATABASE	Search Strategy
PubMed	"rectal stimulation" AND ("Infant, Newborn" [Mesh] OR "newborn" OR "premature" OR "preterm")
CINAHL	"rectal stimulation" AND ("newborn" OR "premature" OR "preterm")
Cochrane	"rectal stimulation" AND ("newborn" OR "premature" OR "preterm")
Embase	'rectal stimulation' AND ('newborn'/exp OR 'newborn' OR 'prematurity'/exp OR 'prematurity')
Scopus	"rectal stimulation" AND ("newborn" OR "premature" OR "preterm")
Web of Science (WoS)	"rectal stimulation" AND ("newborn" OR "premature" OR "preterm")
