# Supplementary material for: Rectal Stimulation in Premature and Full-Term Newborns: A Narrative Review
Source: Children (Basel). 2025 Dec 6;12(12):1656. doi: 10.3390/children12121656 (PMC12731925; doi:10.3390/children12121656)
Supplement: Supplementary file 1 [file children-12-01656-s001.zip › Supplementary File S3.pdf]

Supplementary File S3: PRISMA 2020 flow diagram for new systematic reviews which included searches of databases and registers only

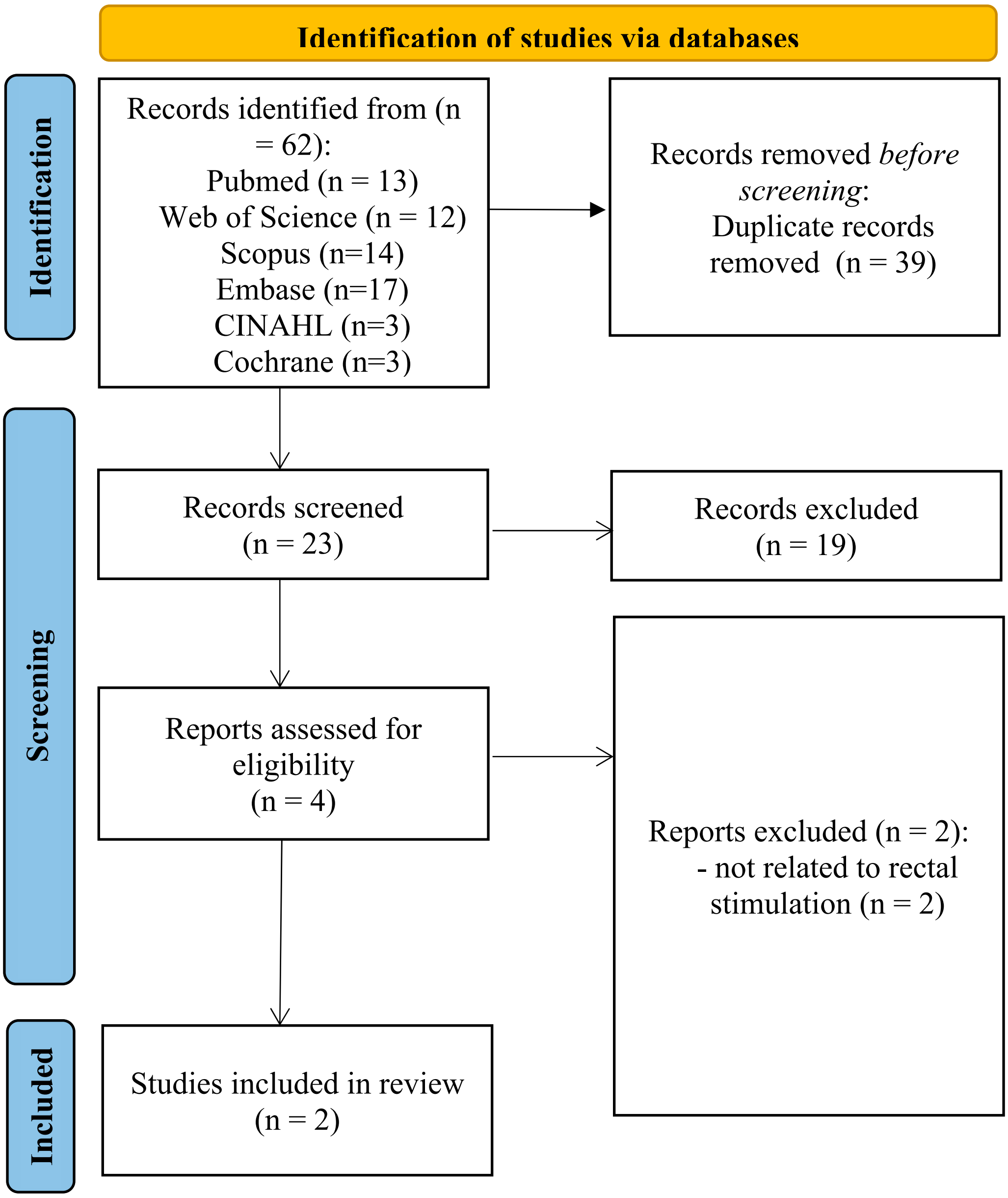

From: Page MJ, McKenzie JE, Bossuyt PM, Boutron I, Hoffmann TC, Mulrow CD, et al. The PRISMA 2020 statement: an updated guideline for reporting systematic reviews. BMJ 2021;372:n71. doi: 10.1136/bmj.n71

For more information, visit: http://www.prisma-statement.org/
